# The evolving cobweb of relations among partially rational investors

**DOI:** 10.1371/journal.pone.0171891

**Published:** 2017-02-14

**Authors:** Pietro DeLellis, Anna DiMeglio, Franco Garofalo, Francesco Lo Iudice

**Affiliations:** Department of Electrical Engineering and Information Technology, University of Naples Federico II, Naples, Italy; Georgia Institute of Technology, UNITED STATES

## Abstract

To overcome the limitations of neoclassical economics, researchers have leveraged tools of statistical physics to build novel theories. The idea was to elucidate the macroscopic features of financial markets from the interaction of its microscopic constituents, the investors. In this framework, the model of the financial agents has been kept separate from that of their interaction. Here, instead, we explore the possibility of letting the interaction topology emerge from the model of the agents’ behavior. Then, we investigate how the emerging cobweb of relationship affects the overall market dynamics. To this aim, we leverage tools from complex systems analysis and nonlinear dynamics, and model the network of mutual influence as the output of a dynamical system describing the edge evolution. In this work, the driver of the link evolution is the relative reputation between possibly coupled agents. The reputation is built differently depending on the extent of rationality of the investors. The continuous edge activation or deactivation induces the emergence of leaders and of peculiar network structures, typical of real influence networks. The subsequent impact on the market dynamics is investigated through extensive numerical simulations in selected scenarios populated by partially rational investors.

## Introduction

An interesting debate is taking place in the scientific community on a possible paradigm shift from neoclassical economics [1]. The global economic crisis of 2008 was another evidence of the incompleteness of the existing economic and financial models, which proved incapable of providing warnings and explaining the deepest causes of the crisis [2, 3]. This fact further spurred the interest of other scientific communities, whose contributions were welcomed by the economists, as it is becoming a common belief that a thorough understanding of the intricate dynamics taking place on financial markets requires the integration of tools and perspectives from different disciplines [1, 4].

A key assumption of classical economic and financial models was the rational behavior of the *homo oeconomicus*, but the real markets are inhabited by *common people*. Psychological studies show that the decision-making process, which is the determinant of financial dynamics, cannot be described as perfectly rational [5]. Indeed, it is imperfect due to the presence of uncertainties, approximation errors, emotions, and cognitive biases. Inspired by the early concepts of the *Prospect Theory* [6, 7], and, thanks to the collaborative work of economists, psychologists and sociologists, a new discipline, *behavioral finance*, was born with the goal of investigating the reasoning patterns of the financial agents ultimately unraveling their mental and emotional processes and the way they mutually influence their trading strategies [8]. A pressing open problem is the development of quantitative models capable of translating the principles of behavioral finance into helpful instruments that may inform policy makers, see for instance [9]. Another community that showed remarkable interest in the analysis of financial markets was that of physicists, who looked at them as examples of complex systems that can be studied through the tools of statistical physics [10–12]. Indeed, a novel discipline, econophysics, was born in 1995 [13] and tried to elucidate the macroscopic emerging features of financial markets from the behavior of its micro constituents, i.e the financial agents. Using tools from agent-based modeling [14–17], artificial financial markets were developed to reproduce and explain the so-called *stylized facts* observed in real markets [14, 18–26]. For instance, in [23] the authors showed how scaling in finance arises from mutual interactions of market participants, while in [24] a realistic trading mechanism for price formation is reproduced. The study of financial markets represents an intriguing challenge for the engineering community as well, which also started to contribute in this field, see for instance [24, 26, 27] and references therein.

We wish to remark that, even though the effort of several scientific communities is producing noticeable work that is clarifying certain aspects of the market fluctuations, a thorough understanding of the cause-effect relationship among the agents’ behavior, decision of policy makers, and market dynamics is still missing. One of the unanswered questions is the impact of the cobweb of relationship among the agents on the market evolution. Indeed, it is well known that social influence biases individual decision making [28]. In the literature, social influences have been often modeled through interaction networks that are either considered static [29], time varying according to the rate of transmission of information [30], or randomly generated at each iteration [31]. However, in real markets the influence among the agents may be dynamic [32, 33] thus determining an adaptive topology whose evolution may be driven, for instance, by the perceived successfulness of the agents, with some central nodes of the network loosing their leadership in favor of other agents that are climbing the market [34–36]. State-dependent probabilistic laws have been used to couple the evolution of the agents’ state with network dynamics in socioeconomic phenomena, such as the diffusion of trust or technological expertise [37, 38]. Differently form the existing literature, we model network evolution in financial markets though the dynamical systems paradigm, so as to reproduce the effect of memory in social dynamics [39]. Specifically, we employ the edge snapping mechanism, firstly introduced in [40] to model network evolution in complex networks, and to describe the variable patterns of influence that determine the spontaneous election and decline of leading investors. In particular, we illustrate how leadership emerges in presence of different degrees of the investors’ rationality and explore the impact on macroscopic observables, such as the wealth distribution and the overall transaction volumes, by means of a thorough numerical analysis.

## Methods

**Fig 1:**
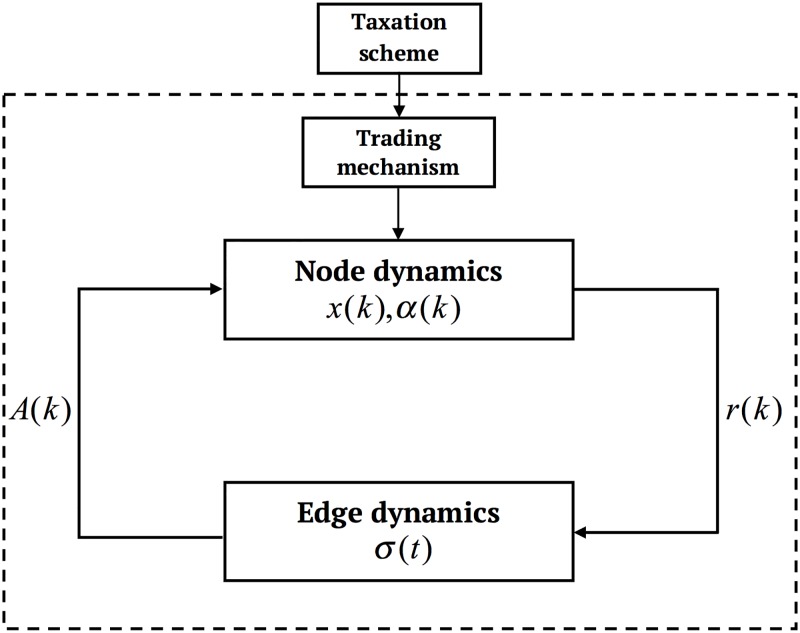
Schematic of the investment market.

Leveraging tools from agent-based modeling and complex networks theory, we model the investment market as an evolving dynamical network [41], where the node state variables describe the current wealth and investing attitude of each financial agent, while the edge state variables determine the dynamical evolution of the cobweb of influence relationship among the agents. A schematic of the investment market is given in Fig 1. The state of each agent is described by its current wealth *x* and risk attitude *α*. In turn, the agent reputation is evaluated as a function of its state and triggers the edge dynamics, which produce the time-varying adjacency matrix *A*(*k*). The latter describes the current influence among the agents and is fed back to the node dynamics. In what follows, we describe in details node and edge dynamics, and then focus on the forces driving network evolution.

### Node dynamics

Let us consider a market populated by *n* financial agents, which may be individuals or corporations. The state of the *j*-th agent at the beginning of the *k*-th trading session is represented by a financial and a behavioral variable, that is, its current wealth *x*_*j*_(*k*), and its current risk attitude *α*_*j*_(*k*), respectively. In particular, the wealth at time *k* depends on the wealth at *k* − 1, and on the agent investing strategy *τ*_*j*_. Namely,

$$\begin{array}{c} x_{j}\left(k\right)=\phi (x_{j}\left(k-1\right),\tau_{j}\left(\alpha_{j}\left(k-1\right)\right)), \end{array}$$ (1)

where the function *ϕ* accounts for the specific structure of the market. Every agent selects its investing strategy *τ*_*j*_(*k* − 1) on the basis of its risk attitude *α*_*j*_(*k* − 1), which is updated as follows:

$$\begin{array}{c} \alpha_{j}\left(k\right)=\left\{\begin{array}{cc} \left(1-w\right)\alpha_{j}\left(0\right)+\frac{w}{\left|N_{j}\left(k-1\right)\right|}\sum_{i=1}^{N}a_{ij}\left(k-1\right)\alpha_{i}\left(k-1\right), & \text{if}\left|N_{j}\left(k-1\right)\right|>0\\ \alpha_{j}\left(0\right) & \text{otherwise} \end{array}\right. \end{array}$$ (2)

for all *j* = 1, …, *n*, where 0 ≤ *w* ≤ 1 is an interaction weight, $\alpha_{j}\left(0\right)=\alpha_{j}^{o}$ is the innate risk attitude of the agent, *a*_*ij*_(*k*) is the element (*i*, *j*) of the adjacency matrix *A*(*k*) defining the time-varying influence network, which will be described in the next section, and $N_{j}\left(k\right):=\{i:a_{ij}\left(k\right)=1\}$ is the set of neighbors of node *j* at time *k*. We remark that the interaction topology is directed, namely the existence of the link (*i*, *j*) does not imply the existence of the link (*j*, *i*).

During the trading sessions, the *reputation* of each agent *r*_*j*_(*k*), which is a time-varying attribute conferred to *j* by the *other* agents, is built. To avoid an overly complex modeling, we consider the reputation of the agent independent from the agent assessing it. Indeed, a relaxation of this assumption does not impact on the results shown in this work, see S1 Supporting Information. Here, the agent reputation is computed as a convex combination of its current wealth, that is a measure of the effectiveness of its trading history, and the intensity *c*_*j*_ of its *charisma*, which is a personal quality that magnifies the capability of influencing its peers independently from its trading skills. Namely,

$$\begin{array}{c} r_{j}\left(k\right)=\left(1-\nu \right)x_{j}\left(k\right)+\nu c_{j},\;j=1,\ldots ,n, \end{array}$$ (3)

where 0 ≤ *ν* ≤ 1 is the *irrationality coefficient* that quantifies the extent of irrationality permeating the market. Following [42], in this work we call the market irrational when the agents fail to correctly evaluate the trading abilities of their peers, and start being influenced by incompetent but charismatic peers. We emphasize that, as the level of irrationality in the market varies depending on the coefficient *ν*, the reputation of an agent may be more or less influenced by the intensity of its charisma.

### Edge dynamics

To mimic the variable patterns of aggregation observed in financial markets [42], at every trading session, edges between agents can emerge or disappear. Namely, the topology of the influence network among the agents can evolve depending on the relative agent reputations. Typically, each agent cannot interact with all the others: in real social networks the interaction mechanism is selective and not all-to-all, as individuals have a finite communication capacity [43–46]. Accordingly, we introduce the graph $P=\{V,E_{p}\}$ defining the *social capacity* of every agent, where V is the set of agents, and $E_{p}$ is the set of edges (the relations) that can be activated.

**Fig 2:**
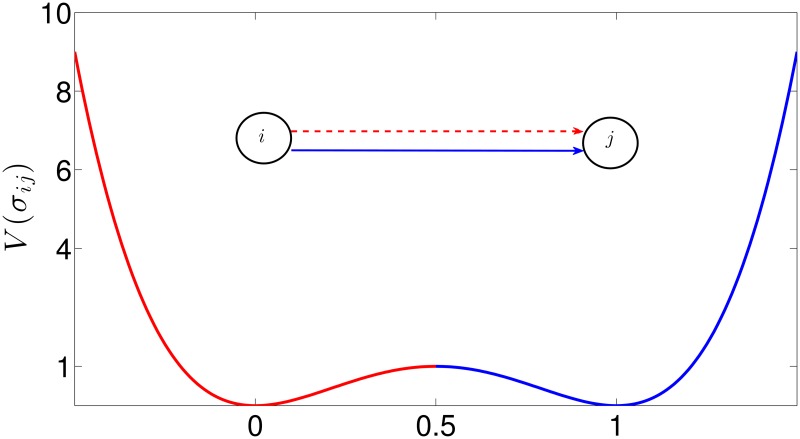
Potential driving the edge evolution with *b* = 16. The red dotted arrow corresponds to an inactive edge, while the blue solid arrow to an active one.

To capture the evolutive dynamics of the mutual influence among financial agents, we establish an analogy between the edge activation/deactivation and the motion of a mass in a double-well potential. Being closer to the first (second) well determines the edge to be active (inactive). In formal terms, the activation or deactivation of an edge $\left(i,j\right)\in E_{p}$ depends on the value of the state variable $\sigma_{ij}\in R$ associated to each potential edge in the network. Leveraging the edge snapping mechanism proposed in [40], we model the edge evolution through the following set of differential equations:

$$\begin{array}{c} {\ddot{\sigma }}_{ij}\left(t\right)+d{\dot{\sigma }}_{ij}\left(t\right)+\frac{dV(\sigma_{ij}\left(t\right))}{d\sigma_{ij}\left(t\right)}=u_{ij}\left(r_{i}\left(\left\lfloor t\right\rfloor \right),r_{j}\left(\left\lfloor t\right\rfloor \right)\right), \end{array}$$ (4)

for all $\left(i,j\right)\in E_{p}$, where *d* is a damping parameter, *V* is a bistable potential, and $u_{ij}:R\times R\to R$ is a driving force, which is a function of the reputation of the agents’ pair. The bistable potential V:R→R is

$$\begin{array}{c} V\left(\sigma_{ij}\right)=b\sigma_{ij}^{2}{\left(\sigma_{ij}-1\right)}^{2}, \end{array}$$ (5)

where *b* sets the height of the barrier separating the two equilibrium points, see Fig 2.

The edge dynamics determine the adjacency matrix *A*(*k*) describing the active edges at time *k*. Specifically, its element *a*_*ij*_(*k*) is computed as follows:

$$\begin{array}{c} a_{ij}\left(k\right)=\left\{\begin{array}{cc} 1 & \text{if}\;\left(i,j\right)\in E_{p}\;\text{and}\;\sigma_{ij}\left(k\right)>0.5\;\\ 0 & \text{otherwise} \end{array}\right. \end{array}$$ (6)

Indeed, at time *k*, the edge (i,j)∈P is *active* if *σ*_*ij*_(*k*) > 0.5, while it is *inactive* otherwise, as illustrated in Fig 2. The time varying matrix *A*(*k*) is associated to the graph G(k)={V,E(k)} defining the *influence network* among the agents. Namely, (*i*, *j*) belongs to $E\left(k\right)\subseteq E_{p}$ if *a*_*ij*_(*k*) = 1. Notice that the update of *A*(*k*) (and then of G(k)) has a direct influence on the node dynamics, see Eq (2) and Fig 1.

The drivers of the edge evolution are embedded in the function *u*_*ij*_ in Eq (4), whose shape may vary depending on the extent of irrationality in the market, as explained in the following.

### Driving forces

There are multiple drivers determining the emergence and/or the dissolution of an influence relation between financial agents [47]. Here, we assume that the link activation (or deactivation) depends on the relative reputation among the financial agents defined in Eq (3). Accordingly, the force driving the edge evolution is selected as follows:

$$\begin{array}{c} u_{ij}\left(k\right)={\left(-1\right)}^{a_{ij}\left(k\right)}\max\{0,{\left(-1\right)}^{a_{ij}\left(k\right)}(r_{i}\left(k\right)-r_{j}\left(k\right))\}, \end{array}$$ (7)

for all $\left(i,j\right)\in E_{p}$. In simple words, the absence of the edge (*i*, *j*) at time *k* that is, *a*_*ij*_(*k*) = 0, implies that agent *i* does not influence agent *j*, although agent *i* belongs to the social network of agent *j*. In that case, an input *u*_*ij*_(*k*) = *r*_*i*_(*k*) − *r*_*j*_(*k*) may induce the activation of the edge (*i*, *j*) in a future trading session only if the reputation of *i* is higher than that of *j* (*r*_*i*_ > *r*_*j*_). Symmetrically, if *a*_*ij*_(*k*) = 1, an edge may be deactivated only when *r*_*j*_ > *r*_*i*_. We emphasize that the edge activation or deactivation is not instantaneous, as it is filtered by the dynamical system Eq (4), which introduces inertia. This mimics the effect of memory in social dynamics [39]: a significant difference of reputation has to persist for a sufficient time-span to determine a variation in the network topology.

Looking at Eqs (3) and (7), we notice that by varying the value of *ν* in Eq (3), we can move on what we call the *spectrum of market rationality*: its origin corresponds to a market populated by agents behaving as the *homo oeconomicus* (*ν* = 0), while at the end of the spectrum the agents are solely inspired by their subjective perceptions (*ν* = 1). Indeed, in a perfectly rational market, the relative reputation is measured by the wealth difference (*r*_*i*_ − *r*_*j*_ = *x*_*i*_ − *x*_*j*_), which becomes the only driver of the edge evolution through Eq (7). When irrationality dominates the market, the different intensities of the agents’ charisma (*r*_*i*_ − *r*_*j*_ = *c*_*i*_ − *c*_*j*_) determine the edge evolution. We emphasize that low values of the irrationality coefficient could trigger a potentially virtuous phenomenon of rational adaptation, in which the agents tend to account for the investing strategies of the most wealthy investors. On the other hand, as irrationality pervades the market, the agents start to follow charismatic leaders irrespectively of the trading outcome, a scenario that we call irrational herding [48].

### Trading mechanism and taxation

Following the work in [49], we focus on a simplified competitive market where the agents can choose to invest on a set of alternative portfolios of financial assets, characterized by a limited availability and different expected return and volatility. Specifically, at every trading session, an agent, say *j*, based on its current risk attitude *α*_*j*_(*k*), selects the portfolio *ℓ*_*j*_(*k*) to which it currently associates the highest expected utility, and invests in it a fraction *δ* of its current wealth (see S1 Appendix for details). Moreover, the market is regulated by a taxation scheme that redistributes the wealth while keeping its total unchanged. Hence, the generic wealth dynamics in Eq (1) become

$$\begin{array}{ll} x_{j}^{-}\left(k\right) & =x_{j}\left(k-1\right)+\beta_{j}\left(k\right)\delta x_{j}\left(k-1\right)\left(a_{l_{j}\left(k\right)}-1\right)\\ & -\left(1-\beta_{j}\left(k\right)\right)\delta x_{j}\left(k-1\right)\left(1-b_{l_{j}\left(k\right)}\right), \end{array}$$ (8)

$$x_{j}\left(k\right)=\chi \left(x_{j}^{-}\left(k\right)\right),$$ (9)

where *a*_*ℓ*_*j*_(*k*)_ and *b*_*ℓ*_*j*_(*k*)_ are the win and loss rates associated to portfolio *ℓ*_*j*_(*k*), respectively, *β*_*j*_(*k*) is a realization of a uniform Bernoulli random variable *B* describing the output of the trade, and *χ* is a function describing the considered taxation scheme.

## Results and discussion

The potential impact of the edge dynamics on the market evolution is threefold: i) a first direct impact is on the topological structure of the influence network G(k); ii) then, the variation of G(k) (and therefore of the associated adjacency matrix *A*(*k*)) induces a variation of the dynamics of the risk attitudes, as from Eq (2); iii) finally, the change of the risk attitude *α* may indeed have an impact on the investing strategy *τ*_*j*_, thus affecting the wealth distribution across the agents, see Eq (1). Here, we investigate these three effects by means of a thorough numerical analysis, which focuses first on the case of rational adaptation (*ν* = 0), and then accounts for the presence of irrationality.

### Numerical set up

We consider an artificial investment market populated by *n* = 1000 agents with average wealth $\bar{x}=100$. At each trading session, they can choose among three alternative portfolios of investments. The agents are grouped in three classes (of equal size) depending on their innate risk attitudes, which are uniformly distributed in the interval [0.5, 1] as in [49]. Namely, they are classified as audacious if $\alpha_{j}^{o}\in \left[0.83,1\right]$, ordinary if $\alpha_{j}^{o}\in \left[0.67,0.83\right)$, and prudent otherwise. These three agent classes are chosen so that the prudent agent will only consider investing in the less risky portfolio, the ordinary will also consider the averagely risky portfolio, while the audacious agents will invest in the riskiest one as well. The selected taxation scheme is Tobin-like, see S1 Appendix for further details. Also, we set a poverty threshold *x*_*p*_ = 20, below which the agents do not invest and exploit the redistributive effect of the tax. We stress that the poor agents’ wealth is perceived as negligible by its neighbors when evaluating its reputation, with Eq (3) becoming *r*_*j*_(*k*) = *νc*_*j*_.

As for the parameters defining the edge dynamics, the intensity of the agents’ charisma is randomly selected from an exponential distribution of parameter λ = 1. The mean of this distribution is amplified of a factor 100 to coincide with the expected value of the wealth: in this way, the share of reputation determined by the charisma is given by the irrationality parameter *ν*. We point out that the results illustrated in what follows also hold for alternative charisma distributions, such as the uniform and the Gaussian ones, see S1 Supporting Information for further details. As for the social capacity topology P, it is randomly generated by applying a degree-preserving rewiring algorithm to a nearest neighbor graph with average degree 〈*k*〉 = 52. The impact of the snapping dynamics on the market will be tested for increasing values of the irrationality coefficient *ν*. In this context, the extent of rationality in the market refers to the way the agents evaluate their reputation. Accordingly, we select *ν* = 0 and *ν* = 1 to model the purely rational and irrational investment markets, respectively, while we choose *ν* = 0.75 as a representative example of partially rational market. We randomly select initial conditions for all the *σ*_*ij*_ such that $\left(i,j\right)\in E_{p}$ and let the investment market evolve for a sufficient time span (*T* = 15000 trading sessions) so that a steady-state wealth distribution is achieved. To isolate the effect of the snapping evolution from that of other possible drivers, as for instance the selected taxation scheme, we evaluate the results against two reference scenarios: i) a market with non-interacting agents and ii) a market where the interaction is triggered on an Erdös and Rènyi (ER) random undirected topology [50]. All the results reported below are averaged over 100 repetitions for each value of *ν*.

### Rational market evolution (*ν* = 0)

In what we called a perfectly rational market, subjective factors like the agent charisma should not affect the edge evolution. We model this scenario by setting the irrationality coefficient *ν* to zero so that the reputation of each agent is solely determined by its current wealth, that is, *r*_*j*_(*k*) = *x*_*j*_(*k*). In what follows, we explain the effect of perfect rationality on market dynamics.

#### Impact on the network topology

In a perfectly rational market, the reputation of the agents, which drives the edge dynamics, is quantified by an objective and measurable variable, that is, their wealth. As the agents’ wealth persistently changes, because of the stochastic nature of the investment outcome (see the variable *β*_*j*_(*k*) in Eq (8)), the network topology will persistently vary along the trading sessions. To quantify these variations, we defined the *network variability* *η*(*k*) as the fraction of potential edges activated or deactivated at every session, that is,

$$\eta \left(k\right):=\frac{{∥A(k)-A(k-1)∥}_{1}}{\left|E_{p}\right|}.$$

**Fig 3:**
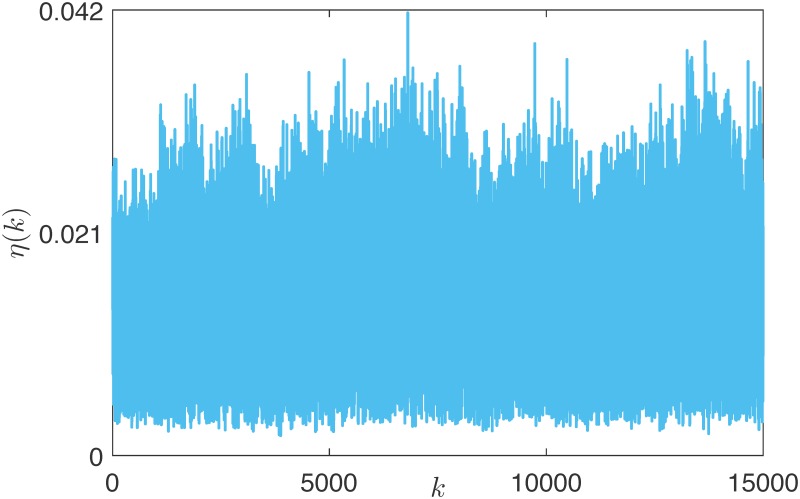
Network variability *η*(*k*) in the rational market.

As illustrated in Fig 3, *η*(*k*) is persistently greater than zero at every trading session, with an average value of 0.01.

**Fig 4:**
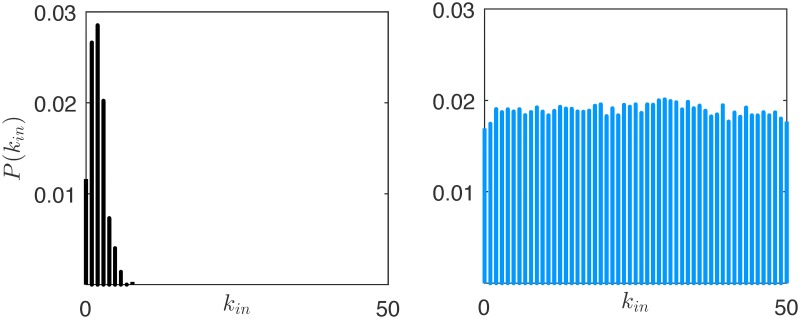
Indegree distribution *P*(*k*_*in*_) of the network in the rational market at *k* = 1 (a) and at *k* = 15000 (b).

**Fig 5:**
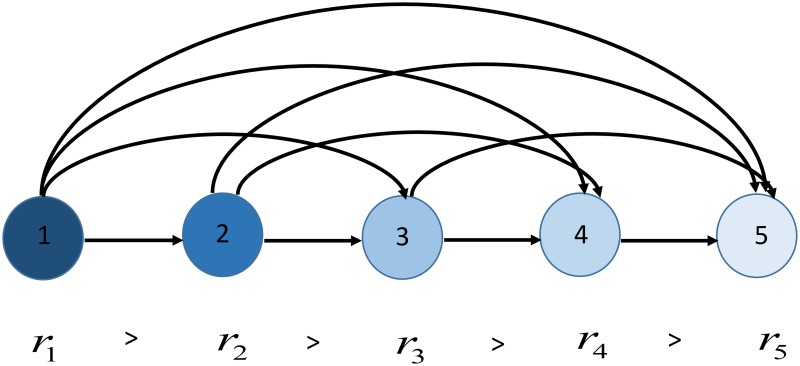
Example of graph with uniform indegree and outdegree distributions.

However, while the network continues to change, some topological properties remain almost unchanged throughout the evolution. For instance, it is interesting to discuss the steady-state distribution of the indegree (similar considerations hold for the outdegree distribution). At the onset of the network evolution, as the initial conditions are randomly selected, the indegree distribution is Poisson-like, see the left panel of Fig 4. Then, after a transient, the indegree distribution settles, and, averaging the distribution in the 100 simulations, we observe an almost uniform distribution in the interval [0 52], right panel of Fig 4. This distribution shows striking similarities with the degree distribution of the corporate elite network in the US, which was also shown to be close to the uniform [51]. A possible explanation of this common behaviour is that in networks of influence, like the one considered in this work or the real corporate elite network studied in [51], the nodes are ranked based on what we call, in this paper, reputation, and the links almost always point from nodes with a higher reputation (the *influencers*) to nodes with a lower reputation (the *followers*). In case this unwritten rule were always followed, and every link could be in principle activated, a perfectly uniform degree distribution would be obtained, as for the graph illustrated in Fig 5. However, in real influence networks, this rule is less compelling, and the interaction is selective, that is, not every link in the network may be activated [44], thus leading to a moderate deviation from a perfectly uniform distribution. Our edge snapping mechanism is capable of reproducing this second, and more realistic, degree distribution. Indeed, the topology is not instantly updated, as its evolution is filtered by the dynamical system Eq (2), which adds inertia to the activation or deactivation of links. Therefore, a higher reputation of node *i* compared to that of node *j* implies a higher likelihood of edge (*i*, *j*) compared to (*j*, *i*), but does not guarantee its activation. In combination with the selective interaction due to limited social capacity of the agents, this allows the model to display moderate deviations from a uniform distribution, thus making it closer to a real influence network.

**Fig 6:**
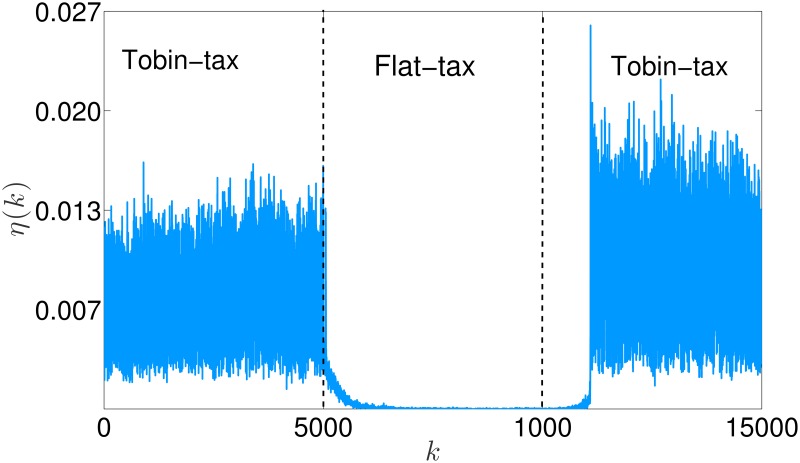
Network variability *η*(*k*) in the rational market under variable taxation schemes.

As the degree distribution is determined by the snapping mechanism, a question naturally arises: what is the cause of the persistent network variability shown in Fig 3? We argue that the variability of the network topology is an indirect measure of the chances that the wealth ranking among the agents changes. Indeed, due to the stochastic nature of the investments and the redistributive effect of Tobin-like taxation schemes [52], the poorest nodes may increase their wealth, thus *climbing* the pyramidal network structure: in the limit example of Fig 5, one or more nodes climbing the market would only correspond to a relabeling of the nodes, but would have no effect on the network structure. Different market structures, which would translate into different shapes of the function *ϕ* in Eq (1), may hinder agent recovery from poverty, thus reducing the network variability. A striking example can be obtained by considering the impact of a less fair taxation scheme. For instance, we report in Fig 6 the outcome of a single run of the market simulation, in which at time 5000 the taxation scheme is changed to a flat tax (see S1 Appendix for details on this scheme), and then is switched back to a Tobin-like tax at time 10000. Differently from the Tobin-like tax, the flat tax has no redistributive effect, as the rate of the tax is independent from the agents’ wealth [53]. This dramatically reduces the opportunities for an agent to climb the wealth rankings. Accordingly, the network variability strongly decreases when the flat tax is introduced, and then slowly returns to oscillating in the usual range when the Tobin-like tax is introduced again.

#### Impact on the risk attitudes

The evolution of the influence network has a direct impact on the risk attitude of the agents. Indeed, Eq (2) implies that the risk attitude of the *j*-th agent is updated through a weighted average between its innate attitude and the current average attitude of the set $N_{j}\left(k\right)$ of its neighbors (the set of nodes with outgoing connections towards *j*), if the latter is non-empty. Namely, Eq (2) can be rewritten as

$$\begin{array}{c} \alpha_{j}\left(k\right)=\left\{\begin{array}{cc} \left(1-w\right)\alpha_{j}\left(0\right)+\frac{w}{\left|N_{j}\left(k-1\right)\right|}\sum_{i\in N_{j}\left(k-1\right)}\alpha_{i}\left(k-1\right), & \text{if}\left|N_{j}\left(k-1\right)\right|>0\\ \alpha_{j}\left(0\right) & \text{otherwise} \end{array}\right. \end{array}$$ (10)

**Fig 7:**
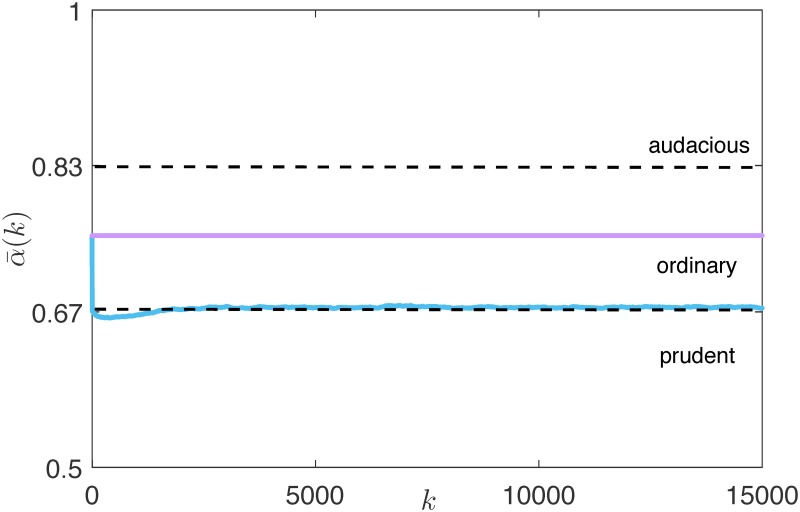
Average risk attitude $\bar{\alpha }\left(k\right)$ of the network in the reference scenarios (magenta line) and in the rational market (blue line).

**Fig 8:**
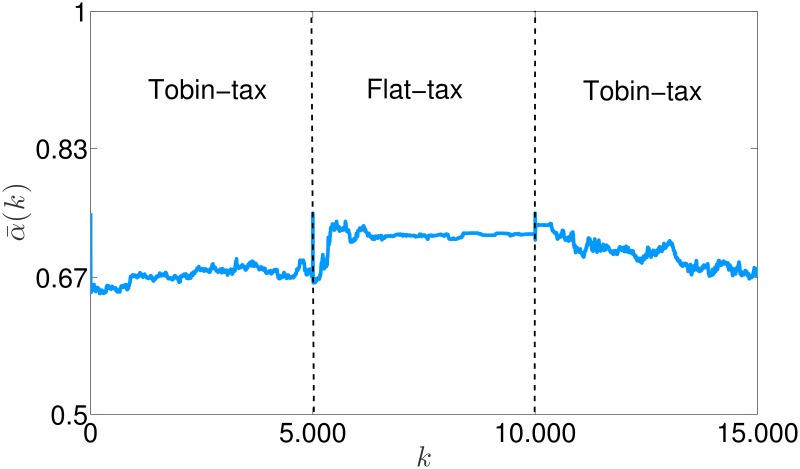
Average risk attitude $\bar{\alpha }\left(k\right)$ in the rational market under variable taxation schemes.

Therefore, as the edge states evolve, *a*_*ij*_(*k*) is updated, with the effect of a persistent variation of the set $N_{j}\left(k\right)$, which in turn implies that risk attitude dynamics never settle. Moreover, we observe that the average risk attitude decreases if compared with the case of no interaction among the agents, and with the case of an ER undirected random influence topology, in which it remains constant, see Fig 7. Indeed, in a rational market the reputation is built based only on the agents’ wealth: when a Tobin-like tax is considered, the prudent agents are favored [49], and therefore the edge snapping dynamics steer the agents attitude towards prudence, with the poorest agents trying to emulate the successful strategy of the richest ones. We emphasize that, when *ν* = 0, the snapping dynamics are also capable of adapting to possible variation in the trading mechanism: for instance, we observe that, when the taxation scheme changes, the most effective investing strategy changes, and the risk attitudes start drifting accordingly, see Fig 8. Indeed, when the flat tax, which rewards more audacious traders [49], replaces the original taxation scheme, the average risk attitude starts to increase, with this tendency reversed when the Tobin-like tax is reintroduced.

#### Impact on the wealth distribution

**Fig 9:**
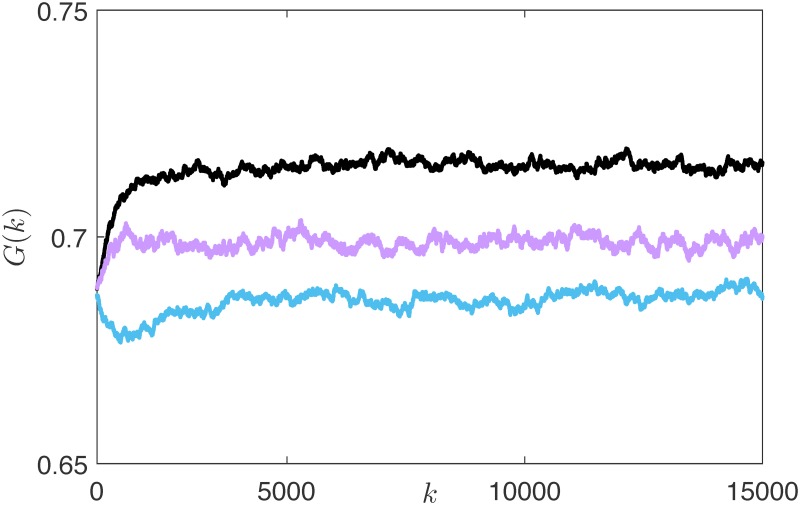
Evolution of the Gini coefficient *G*(*k*) in a market without interaction (black line), in a market with random interaction (magenta line), and in the rational market (blue line).

The modification of the risk attitude induced by the introduction of the snapping mechanism has an impact on the overall dynamics of the market, and in particular on the wealth distribution. To quantify the extent of the inequalities among the agents, we used the Gini coefficient, introduced by Corrado Gini in [54], which can vary between 0 (perfect equality among the agents’ wealth) and 1 (all the wealth belongs to one agent). As expected, because of the learning mechanism, the topological adaptation is beneficial and induces wealth redistribution in the market: from Fig 9 we notice that the Gini coefficient decreases if compared with both the reference scenarios.

### The onset of irrationality (*ν* > 0)

**Fig 10:**
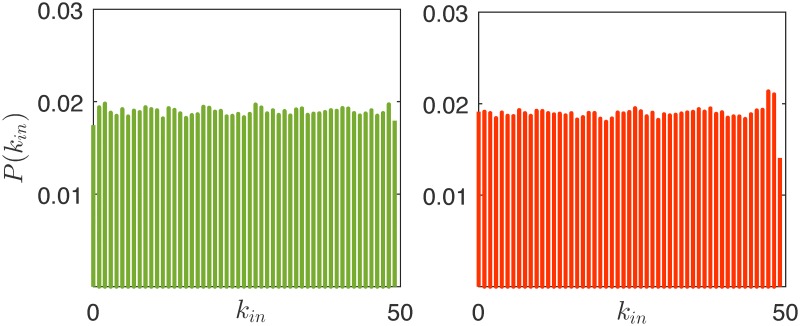
Indegree distribution *P*(*k*_*in*_) in the partially rational market (a) and in the irrational market (b) at *k* = 15000.

**Fig 11:**
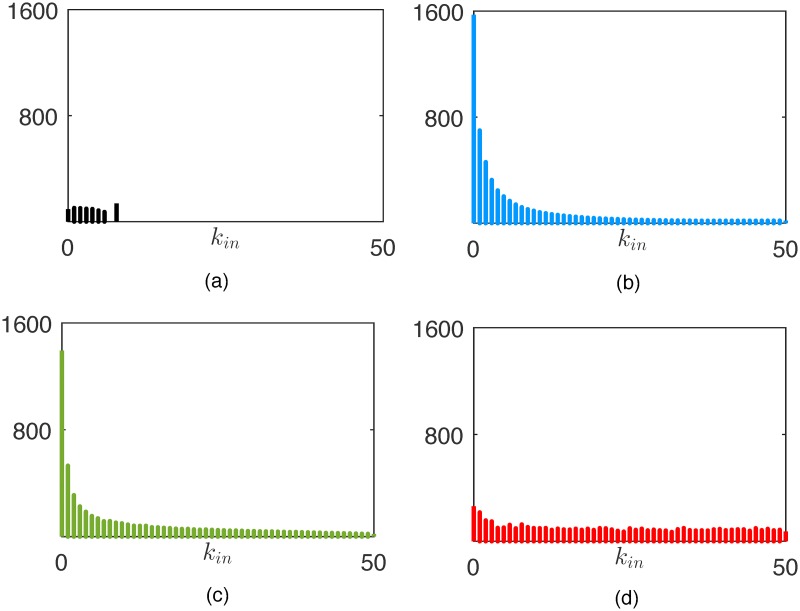
Average wealth of an agent as a function of its indegree in the rational market at *k* = 1 (a) and at *k* = 15000 (b), and in the partially rational market (c) and irrational market (d) at *k* = 15000.

**Fig 12:**
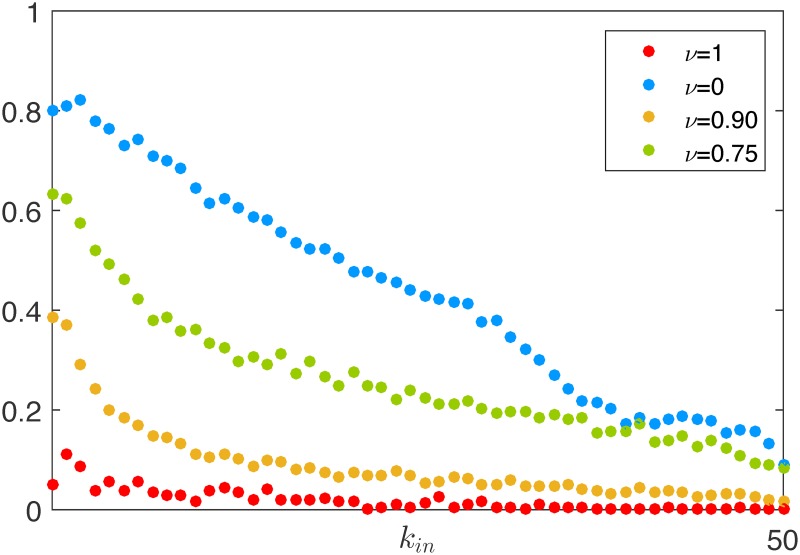
Average fraction of prudent agents as a function of their indegree for different values of *ν* at *k* = 15000.

As irrationality pervades the market, the reputation of each agent becomes more and more influenced by a subjective variable, that is, the innate intensity of its charisma. An analysis of the steady-state degree distribution demonstrates that it is approximately uniform regardless of the level of irrationality in the market, see Figs 4 and 10. Although the structural properties of the graph do not change, the ranking of the nodes in the hierarchical structure of Fig 5 becomes less and less related to the agents’ wealth as the irrationality increases. To clearly illustrate this point, in Fig 11 we report the average wealth of an agent as a function of its indegree (symmetrical considerations hold for the outdegree), and we observe that the dependence between the two quantities becomes weaker and weaker as *ν* gets closer to 1. Indeed, a higher indegree means that the agent is influenced by a large fraction of its neighbors. In the presence of rationality, this happens when it is significantly poorer than its neighbors. This is not the case when irrationality increases. An interesting common denominator across all the levels of irrationality is that the nodes with very low indegree (and high outdegree), tend to have a wealth that is remarkably higher than the average. This can be easily explained in a rational market, in which the edge dynamics are driven by the wealth difference, and then the absence of ingoing links is associated to the richest nodes. When *ν* approaches to one, the explanation is less trivial, and can be obtained by observing that only low indegree agents preserve a relevant fraction of agents with the best (prudent) attitude, see Fig 12. Indeed, the random interaction taking place when *ν* = 1 has the main effect of averaging the attitudes, dramatically increasing the fraction of ordinary nodes. The nodes that are less affected by this effect are the most charismatic, who maintain their initial investing strategies regardless of what the others do. In other words, this means that when irrationality pervades the artificial market, herding is in general of no use, and may also become detrimental.

**Fig 13:**
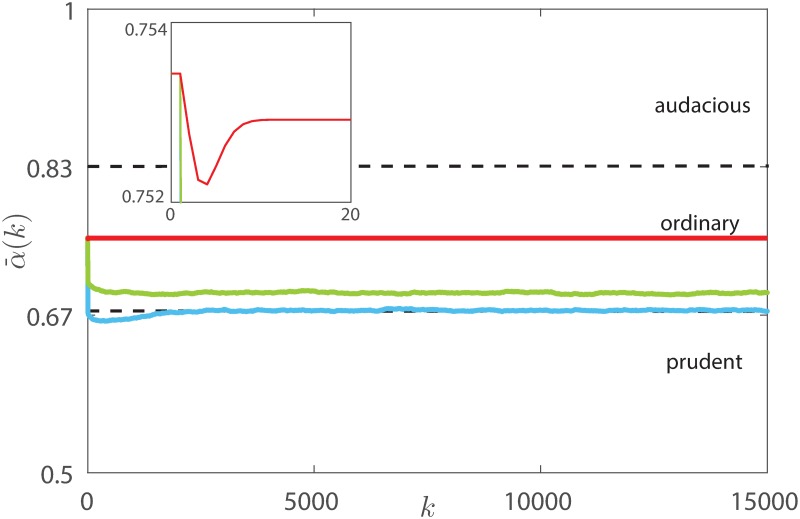
Average risk attitude $\bar{\alpha }\left(k\right)$ in the rational (in blue), partially rational (in green) and irrational (in red) markets.

**Fig 14:**
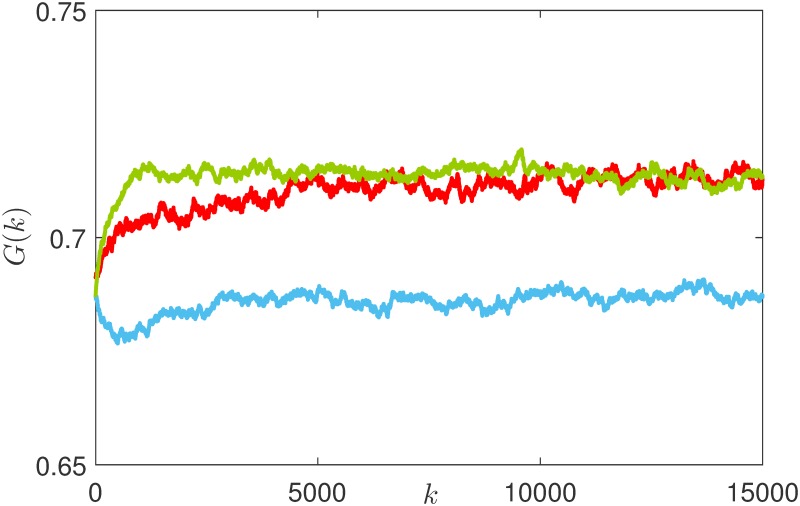
Evolution of the Gini coefficient *G*(*k*) in the rational (blue line), partially rational (green line), and irrational (red line) markets.

**Fig 15:**
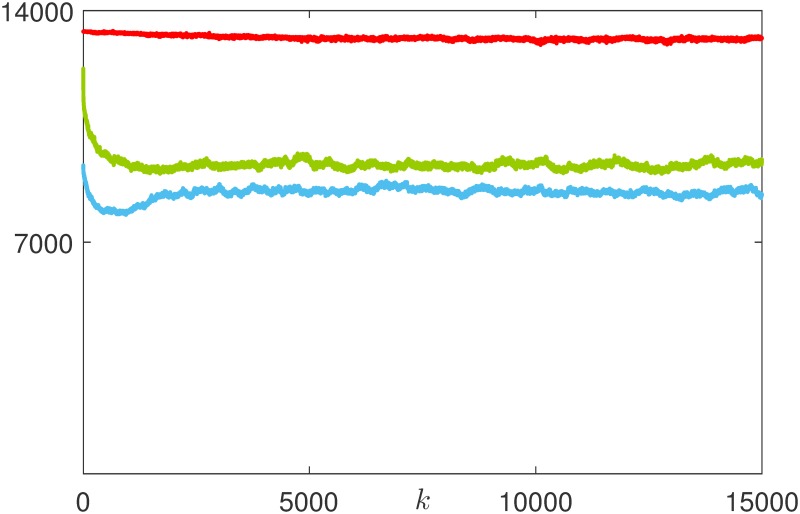
Evolution of the trading volumes in the rational (blue line), partially rational (green line), and irrational (red line) markets.

The reduced rationality also impacts on the investing strategies selected by the agents: compared with the perfectly rational case, the average risk attitude increases and, when *ν* = 1, becomes equivalent to the innate one, see Fig 13. Accordingly, the distribution of the investing strategies is not anymore steered towards the more prudent (and rewarded) ones, and becomes comparable to that obtained with a random undirected ER influence network. Consistently, we observe that the redistributive effect of rational adaptation illustrated in Fig 9 is hampered as irrationality increases, giving place to what we call *irrational herding*, where the potential benefits of the interaction are ruled out by its randomness, see Fig 14. On the other hand, the increased irrationality mitigates one of the known drawbacks of the introduction of Tobin-like tax schemes, that is, the reduction of the trading volumes [52]. Indeed, the irrationality leads to the permanence of a relevant fraction of audacious agents, thus increasing the total volume of trades, see Fig 15.

## Conclusions

In this paper, we explored the interplay between the evolution of the cobweb of relations among financial agents and the overall market dynamics. Taking a new perspective, we exploited the edge snapping mechanism, firstly introduced in [40], to model the coevolution of the influence network with agent dynamics: each link is viewed as a mass moving in a double-well potential, with the first well corresponding to an inactive link and the second to an active one. The driver of link evolution is the relative reputation between possibly coupled agents. Depending on the extent of rationality in the market, the agent reputation may depend more on the its wealth (rationality prevails in the market) or on the intensity of its charisma (irrationality is predominant). Our numerical analyses have shown that:

- The network topology at steady state displays a fairly uniform indegree distribution. This result is due to the fact that the snapping dynamics tend to assign an indegree which is inversely proportional to the agents’ reputation (the opposite happens for the outdegree). This result is consistent with the typical structure of influence networks, in which the agents are ranked based on their reputation, see for instance the network of corporate elite in the US [51].
- The indegree distribution is not significantly affected by the degree of rationality. Indeed, the extent of rationality only impacts on the way the reputation is evaluated and ranked, but not on the network structure. In simple words, the plot of the graph describing the topology remains mostly unchanged, with only the labels identifying the agents reassigned according to the new reputation ranking.
- The rate of the network variability, defined as the number of edges activated or deactivated at each trading session, quantifies the permeability of the market to agents climbing the reputation ranking. Indeed, in a rational market less fair taxation schemes, such as the flax tax, hamper wealth redistribution, thus reducing network variability. As irrationality pervades the market, the reputation is prevalently determined by the agents’ innate charisma, and therefore this also hinders the network variability as modifications of the agents’ wealth have little impact on their reputation.
- Rational adaptation is beneficial for the market stability. Indeed, it favors wealth redistribution and steers the investing strategies towards the most efficient one. Moreover, it confers to the agents the capability of learning from the environment: they react to variations of the market scenario (e.g. changes in the regulations) and adapt their investing strategies accordingly.
- On the other hand, irrational herding fosters inequalities, nullifying the potential benefits of mutual interactions. Indeed, the agents start to follow the strategies of the most charismatic agents, which are not necessarily those with the most effective investing strategies. Interestingly, the nodes with the lowest indegree, that are the charismatic market leaders, who refuse to herd, show a significantly higher average wealth. This means that in an irrational market herding can be detrimental and it is better to be an influencer rather than a follower, which is in accordance with the empirical findings that illustrate how bubbles may appear in conjunction with irrational herding [55].

Coevolving networks have been recently used in social sciences as a useful tool to model situations in which the a feedback mechanism modifies the structure of the network in dependence of the state of the nodes, see for instance [31, 37, 38, 56–60]. On the ground of our numerical results, and supported by the encouraging matching between the outcome of our model, common sense intuition, and a typical influence network, we envision that the coevolving dynamical networks paradigm, might represent a useful tool to generate more realistic models also in the analysis of financial markets. In particular it could turn out to be particularly useful to embody behavioral economic models and investigate the impact of social interaction on market evolution, such as the emergence of leadership and its consequences on global market observables.

## Supporting information

S1 Appendix Trading mechanism and taxation schemes. (PDF)

S1 Supporting Information Supplementary scenarios. (PDF)
