# Supplementary material for: The evolving cobweb of relations among partially rational investors
Source: PLoS One. 2017 Feb 14;12(2):e0171891. doi: 10.1371/journal.pone.0171891 (PMC5308790; doi:10.1371/journal.pone.0171891)
Supplement: S1 Appendix — (PDF) [file pone.0171891.s001.pdf]

The Evolving Cobweb of Relations among Partially Rational Investors

Pietro DeLellis[1*], Anna Di Meglio[1], Franco Garofalo[1], Francesco Lo Iudice[1]

1 Department of Electrical Engineering and Information Technology, University of Naples Federico II, Naples, Italy

* pietro.delellis@unina.it

S1 Appendix - Trading mechanism and taxation schemes

2016

The aim of this appendix is to provide all the details omitted for brevity in the section "Trading mechanism and taxation" of the manuscript. Although they are the same as in reference [1] ([49] in the main manuscript), the details are reported here for the sake of clarity, as the notation used in [1] is slightly different and may confuse the interested reader.

1 Wealth dynamics

The proposed artificial financial market is populated by a set of n agents, who can choose among alternative portfolios. The agents behave according to the Von Neumann and Morgenstern utility theory [2]. At each time step $k = 1, 2, ...,$ a trading session is simulated. Each agent, in a sequential random order, evaluates the convenience of investing a given fraction δ of its current wealth $x_j(k)$ in one of the portfolios from the set $\mathcal{L} = \{\ell_1, \ldots, \ell_m\}$. The portfolios in $\mathcal{L}$ are all characterized by a limited availability $\Upsilon_i, i = 1, ..., m-1$, except the m-th, which is associated to a virtual portfolio, corresponding to no-investment, with $\Upsilon_m = +\infty$. Each agent is allowed to invest in one of the available portfolios, that is, in any element of $\mathcal{L}$ such that $\Upsilon_i \geq \delta x_j(k)$. Agents' access to trading is randomly permuted at each time step k, so that, on average, no agent is favored. After each trading, the availability of the selected portfolio is updated before the next agent is allowed to trade. A power-law utility function characterizes the risk attitude of each agent. At each trading session k, agent j decides to invest in the most profitable portfolio $i \in \mathcal{L}$, selected by comparing the portfolios' expected utilities

$$\mathbb{E}[U_j(x_j(k), i)] = 0.5\left[(a_i \delta x_j(k))^{\alpha_j(k)} + (b_i \delta x_j(k))^{\alpha_j(k)}\right],\ i = 1, ..., m, \tag{S1-1}$$

where $\alpha_j(k)$ is the risk attitude of the j-th agent, a_i and b_i are the win and loss rates associated to the i-th portfolio, $i = 1, \ldots, m$[1]. Namely, at each trading session agent j, based on its risk attitude $\alpha_j(k)$, selects the investment

$$\ell_j(\alpha_j(k)) = \arg\max_{i \in \Lambda} \mathbb{E}[U_j(x_j(k), i)], \tag{S1-2}$$

where $\mathbb{E}$ is the expected value operator, and $\Lambda \subseteq \mathcal{L}$ is the set of portfolios that, in the moment of the trade, have an availability higher than $\delta x_j(k)$. We emphasize here that an agent may decide not to invest (formally, to invest in the m-th portfolio), if $\mathbb{E}[U_j(x_j(k), m)] \geq \mathbb{E}[U_j(x_j(k), i)]$ for all the $i \in \Lambda - \{m\}$.

The outcome of the trade is the realization $\beta_j(k)$ of a uniform Bernoulli random variable B. Therefore, the wealth $x_j^-(k)$ of the agent j at time k before the taxation is

[1] Notice that the win and loss rates associated to the virtual portfolio are $a_m = b_m = 1$.

given by

$$x_j^-(k) = x_j(k-1) + \beta_j(k)\delta x_j(k-1)(a_{\ell_j(k)} - 1) \\ - (1-\beta_j(k))\delta x_j(k-1)(1 - b_{\ell_j(k)}),$$

where we omit the dependence of ℓ_j on $\alpha_j(k)$, and which corresponds to Eq. (8). At this point, the taxation scheme determines the wealth at iteration k as

$$x_j(k) = \chi(x_j^-(k)), \tag{S1-3}$$

which corresponds to Eq. (9). In the next sections, we clarify the taxation mechanism of the Tobin-like and flat taxes considered in the manuscript, that is, we specify function χ.

2 Tobin-like taxation scheme

The Tobin-like tax employed in this work is a financial transaction tax, which reduces the current wealth of the winning agents by a profit fraction $\rho(k)$ given by

$$\rho(k) = \begin{cases} \frac{p(k)}{\sum_{j=1}^n s_j(k)}, & p(k) > 0, \\ 0, & p(k) \leq 0, \end{cases} \tag{S1-4}$$

where $s_j(k) = x_j^-(k) - x_j(k-1)$, and $p(k) = \sum_{j=1}^n (x_j^-(k) - x_{j0})$. Accordingly, Eq. (S1-3) becomes

$$x_j(k) = x_j^-(k) - H(s_j(k))s_j(k)\rho(k), \tag{S1-5}$$

where H is the Heaviside step function. For the sake of brevity, in what follows we refer to this financial transaction tax as Tobin-like Tax (TT).

3 Flat taxation scheme

Adopting a flat tax, the amount of the tax is proportional to the total wealth of the individual. Specifically, it is a non-progressive wealth tax (WT), proportional to the current wealth $x_j^-(k)$ of each agent j, with $j = 1, \ldots, n$. Accordingly, Eq. (S1-3) becomes

$$x_j(k) = \gamma(k)x_j^-(k), \tag{S1-6}$$

where

$$\gamma(k) = \frac{\sum_{j=1}^n x_{j0}}{\sum_{j=1}^n x_j^-(k)}.$$

Notice that, to allow for a proper comparison between the two taxation schemes, the time-varying coefficients $\rho(k)$ and $\gamma(k)$ in Eqs. (S1-4) and (S1-6), respectively, are selected so as to keep the average wealth constant over time, that is,

$$\frac{1}{n}\sum_{j=1}^n x_j(k) = \sum_{j=1}^n x_{j0}.$$

References

1. DeLellis P, Garofalo F, Lo Iudice F, Napoletano E. Wealth distribution across communities of adaptive financial agents. New Journal of Physics. 2015;17(8):083003.

2. Von Neumann J, Morgenstern O. Theory of Games and Economic Behavior (60th Anniversary Commemorative Edition). Princeton University Press; 2007.
