# Supplementary material for: The evolving cobweb of relations among partially rational investors
Source: PLoS One. 2017 Feb 14;12(2):e0171891. doi: 10.1371/journal.pone.0171891 (PMC5308790; doi:10.1371/journal.pone.0171891)
Supplement: S1 Supporting Information — (PDF) [file pone.0171891.s002.pdf]

The Evolving Cobweb of Relations among Partially Rational Investors

Pietro DeLellis[1*], Anna Di Meglio[1], Franco Garofalo[1], Francesco Lo Iudice[1]

1 Department of Electrical Engineering and Information Technology, University of Naples Federico II, Naples, Italy

* pietro.delellis@unina.it

S1 Supporting Information - Supplementary scenarios

2016

Here, we present an additional set of numerical simulations providing evidence of the robustness of our model to the relaxation of some assumptions. In particular, we made several modifications to the reputation function to verify that the main results illustrated in the manuscript still hold.

we have conducted additional numerical analyses modifying the reputation function with the aim of detecting possible failures of the designed influence process and/or discrepancies with our conclusions.

Alternative reputation functions

Noise in the wealth evaluation

We start our robustness analysis by evaluating the impact of adding measurement noise, affecting the agents' evaluation of the wealth of their peers, on the observed evolution of the state variables and of the network. To this aim, for each value of $\nu \in \{0, 0.75, 1\}$, we have performed a set of 10 simulations in which the reputation $r_i(k)$, instead of following Eq. (3), updates according to

$$r_i(k) = (1-\nu)(x_i(k) + \rho) + \nu c_i, \tag{S2-1}$$

where ρ is the measurement noise is selected from a uniform distribution such that the wealth measurement error is between the 2.5% and 5% of its true value. As we can observe from Figs. S2-1 and S2-2, the results presented in the main text still hold as the the qualitative effect on the risk attitude is unchanged and the in-degree distribution remains uniform-like (see Figs. 4, 10, 13 for comparison).

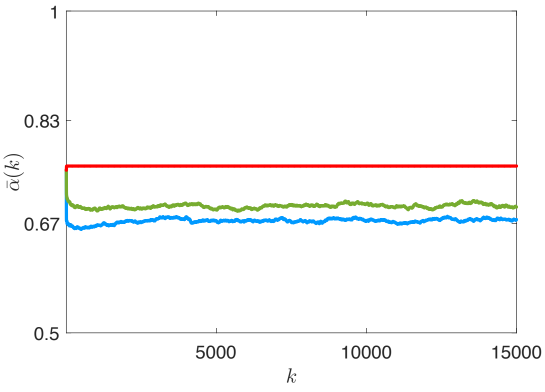

Fig S2-1. Evolution of the agent's risk attitudes when the reputation function is affected by measurement noise.

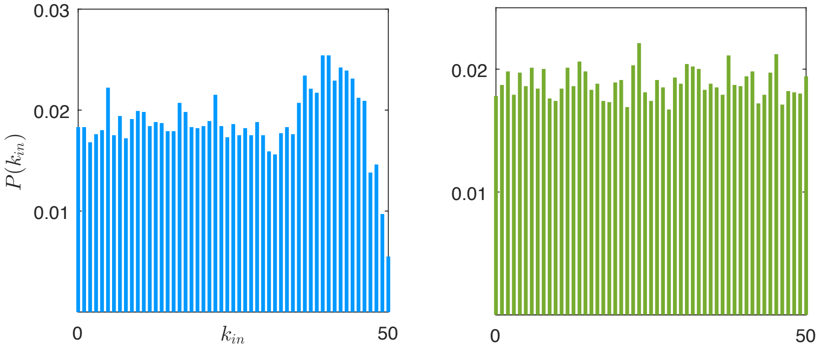

Fig S2-2. In-degree distribution when the reputation function is affected by measurement noise.

Perceived charisma in the reputation function

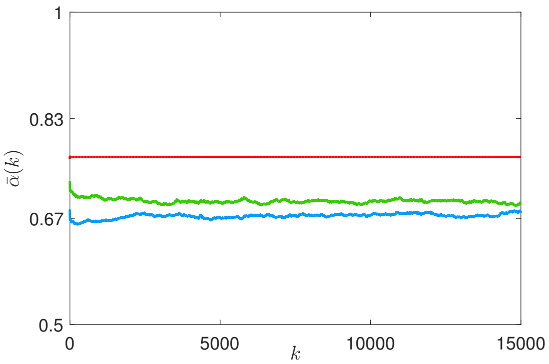

Fig S2-3. Simulation of the artificial market model when the reputation is computed according to Eq. (S2-2). Risk attitude dynamics in the rational (blue) semirational (green) and irrational (red) scenario.

To avoid overly complex modeling, in our work we have assumed that the agents' charisma intensity c_j is independent from the agent assigning it. To provide evidence that this assumption does not impact on our results, we present a set of 10 simulations where each agent j performs its individual evaluation of the charisma of agent i i.e., in general, $c_i^j \neq c_i^l$ $i \neq j$. Namely, Eq. (3) of the main text has been modified as

$$r_i^j(k) = (1-\nu)x_j(k) + \nu c_i^j, \tag{S2-2}$$

where c_i^j, i.e. the charisma intensity of i as perceived by j. Notice that the self-perceptions ($j = i$) of the charisma intensities, c_i^i, $i = 1, \ldots, n$, are randomly selected from an exponential distribution. For all $i = 1, \ldots, n$, $j \neq i$

$$c_i^j = c_i^i + \rho_{ij},$$

where ρ_{ij} is selected from a uniform distribution in $[-0.05c_i^i, 0.05c_i^i]$.[1] As we can observe by comparing Fig. S2-3 with Fig. 13, the impact on the risk attitude is negligible, while Fig. S2-4 shows that the in-degree distribution is still uniform-like.

[1]Notice that in the rational scenario, where $\nu = 0$, the reputation function is unchanged if compared with that of the main manuscript.

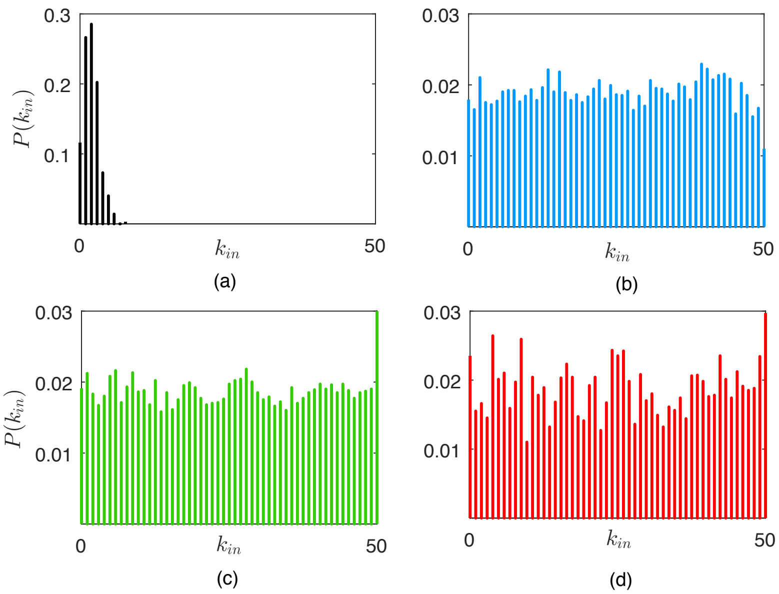

Fig S2-4. Simulation of the artificial market model in the rational scenario when the reputation is computed according to Eq. (S2-2). In-degree distribution in the semirational (a) and irrational (b) scenario.

Derivative-like term in the reputation function

In the main text, the rational component of the agents' reputation is proportional to their current wealth. One may argue that also the rate of wealth change can contribute to building its reputation. Indeed, this would model the case in which the reputation of an agent climbing the market is larger than that of a declining agent when their current wealth is similar. To evaluate the impact of a derivative-like term in the reputation, we have simulated our artificial market replacing Eq. (3) of the main text with

$$r_j(k) = x_j(k) + \gamma\left(x_j(k) - x_j(k-1)\right), \tag{S2-3}$$

where γ determines the relevance of the rate of wealth change of the agents' wealth in determining their reputation. Again, as we may observe from Fig. S2-5, these additional numerical analyses are consistent with the presented results.

Alternative distributions for the charisma intensity

The numerical analyses presented in the main text refer to scenarios in which the agents' charisma is randomly selected from an exponential distribution with parameter $\lambda = 1$. Indeed, it can well be the case that the distribution be different. To evaluate the robustness of our model to variations of the charisma distribution, we have performed two additional sets of simulations, one in which the charisma is selected from a uniform distribution in the interval $[-2, 4]$ and the other one in which it is selected form a normal distribution with mean and variance both equal to 1.[2] Both distributions were tested in the semirational as well as in the irrational scenario. From Figs. S2-6, and S2-7, we can conclude that there are no significant differences among the three distributions.

[2]The mean of all the distributions is amplified of a factor 100 to coincide with the expected value of the wealth: in this way, the share of reputation determined by the charisma is given by the irrationality parameter ν.

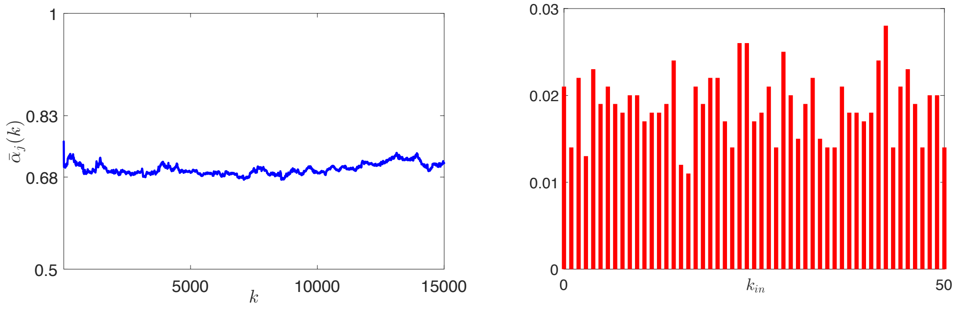

(a) Risk attitude dynamics (b) In-degree distribution

Fig S2-5. Simulation of the artificial market model in the rational scenario when the reputation is computed according to Eq. (S2-3) with $\gamma = 0.75$.

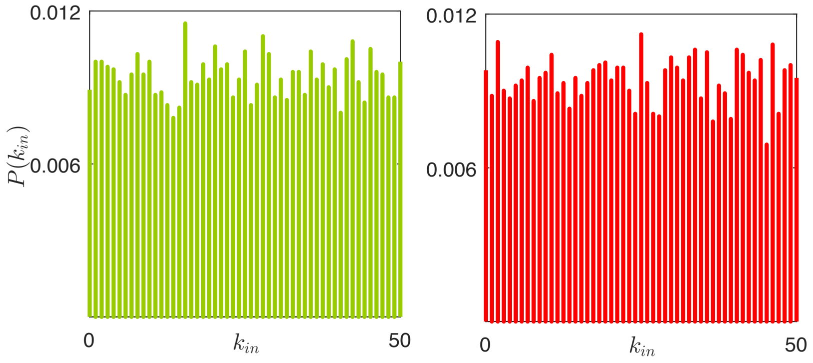

Fig S2-6. In-degree distribution in semirational (in green) and irrational (in red) market with agent charisma generated from a Gaussian distribution. The rational case is not reported as it is independent from the charisma.

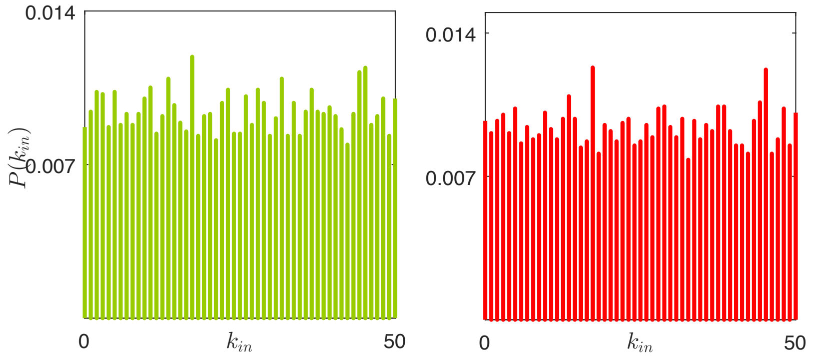

Fig S2-7. In-degree distribution in semirational (in green) and irrational (in red) market with agent charisma generated from a uniform distribution. The rational case is not reported as it is independent from the charisma.
